# Policy implementers’ perspectives of the implementation of the national guidelines for patient safety incident reporting in selected South African public hospitals

**DOI:** 10.1186/s12913-026-14623-x

**Published:** 2026-05-05

**Authors:** Sibongile Dlaba, Nicholin Scheepers, Aaron Asibi Abuosi, Immaculate Sabelile Tenza

**Affiliations:** 1 School of Nursing Science, Faculty of Health Sciences, North West University, Potchefstroom, South Africa; 2 Department of Public Admin and Health Services Management, University of Ghana Business School, Legon, Ghana

**Keywords:** National guidelines, Patient safety incident reporting, Policy implementation

## Abstract

**Background:**

The voices of implementers are crucial in enhancing policy implementation. In the North West Province of South Africa, there have been no studies on the implementation of national guidelines for patient safety incident reporting since its introduction. Hence, this study explored the implementation of the national guidelines for patient safety incident reporting in selected public hospitals in the Dr Kenneth Kaunda district from the implementers’ perspectives.

**Method:**

This study employed a qualitative exploratory design with purposive sampling of hospital leaders, nurses, doctors, physiotherapists, occupational therapists, and pharmacists, leading to a total of 23 focus group discussions, spread across three participating hospitals, with four to seven participants in each focus group. The policy triangle framework of context, content, actors, and process guided the development of the focus group discussion guide and informed a deductive thematic analysis.

**Results:**

Seven themes emerged, including contextual issues leading to implementing guidelines, common incidents & contributing factors, clarity of policy content, actors’ knowledge, role clarity, motivation, and implementation process. The context of implementing guidelines was a need to standardise reporting practices, improve record-keeping, and mitigate potential litigation risks. Key contributing factors to patient safety incidents were inadequate security response, staff shortages, and resource constraints. The content of standard operating procedures was clear, yet lengthy. Discrepancies between the reporting tool and standard operating procedures complicated the reporting process. The actors’ knowledge gaps hindered accurate reporting. Managers lacked effective strategies for motivating their reporting staff, further impeding the system’s efficacy. While intrinsic motivation, grounded in professional accountability, drove some reporting, fears of consequences were present. The process of reporting was considered burdensome, and insufficient feedback mechanisms left staff uncertain about the value of their contributions.

**Conclusion:**

To improve implementation of the patient safety incident reporting, a system-wide approach is necessary; healthcare providers and leaders’ knowledge must be improved, strategies to motivate reporting must be explored, leaders must create environments conducive for reporting, including protection of the reporters, and improvements after every reported system-level weakness are mandatory in order to encourage reporting. If reporting is for learning, anonymous reporting should be emphasised. Reporting processes must be made easy and consider available technology.

**Supplementary Information:**

The online version contains supplementary material available at 10.1186/s12913-026-14623-x.

## Background

Globally, the occurrence of patient safety incidents (PSIs) has become a crisis [1]. Evidence indicates that each year, 134 million PSIs occur in hospitals located in low and middle-income countries (LMIC), leading to 2.6 million fatalities [2]. The reporting of PSIs is acknowledged to be a useful approach to encourage learning from incidents and influence improvement [3, 4]. However, there is limited research on how nationwide policies of PSI are implemented, and how implementers understand and implement these policies.

The World Health Organization (WHO)’s Global Patient Safety Action Plan 2021–2030 encourages the development of national-level policies that guide the improvement of patient safety [5]. In Africa, some countries have established national-level policy guidelines on PSI reporting, reflecting a commitment to patient safety improvement, and on targets set on the global patient safety action plan [6]. However, the recent report on the global patient safety action plan is silent on the country’s experience in implementing nationwide PSI reporting policies [5]. African studies on patient safety have largely focused on patient safety culture [7], identifying cross-cutting challenges such as fear of reporting [8], blame culture in response to reported incidents, and the importance of teamwork in supporting reporting [9]. These studies were not focused on analysing the policy implementation process; studies on the implementation of national guidelines for PSI reporting could contribute shared lessons to other countries pursuing similar implementation. Empirical studies examining the implementation of the national guidelines for PSIs reporting in South Africa are few, and were conducted in the Gauteng and KwaZulu-Natal provinces [10–13] none in the North West province.

South Africa has a two-tiered health system with public & private sector healthcare facilities serving the healthcare needs of the population, 80% of the population receiving healthcare services from the public sector facilities. Public sector facilities are led by the national department of health policies, while private sector facilities each follow their own policies [14]. Strengthening the implementation of policies in the public sector benefits 80% of the population who seek services in these facilities. The reporting of PSIs in the public sector facilities follows a uniform approach as per the national guidelines for PSI reporting, which was first introduced in 2017 and revised in 2022 [15]. The guidelines were developed nationally as a policy that guides reporting in all facilities across all public sector facilities. The hospital unit managers and patient safety committees are expected to contribute to the implementation by translating guidelines into standard operating procedures (SOPs) in their healthcare facility, and by overseeing the implementation, which the frontline healthcare providers are the primary implementers thereof. The guidelines are set to provide guidance on the reporting and, most importantly, on learning from incidents, than just compliance on reporting.

The main problem and research gap leading to this study is that in South African public sector hospitals, although these guidelines exist, reporting remains insufficient, thereby limiting learning from the incidents and improvement [15]. Additionally, a lack of learning from incidents is a contributor to the increased medical litigations [16], which further depletes the already constrained financial resources of the public health sector. A national audit conducted by the Office of Health Standards Compliance (OHSC) in 2020 revealed that public sector healthcare institutions achieved only 35% of reporting PSIs [17]. The report by OHSC meant that there is insufficient reporting of PSIs across the public sector hospitals in the country, including the North West province. The 2024 national annual report on PSIs indicated insufficient reporting and poor quality of data, characterized by incorrect classifications of incidents [18]. Insufficient reporting, in this case is a symptom of a policy implementation gap worth exploring, and implementers are the best source of evidence on how the implementation is ongoing. To contribute knowledge on improving the implementation of national guidelines on PSI reporting, this study aimed to explore the perspectives of implementers (healthcare leaders and healthcare providers) on the implementation of national guidelines for PSI reporting in the selected hospitals in the Dr Kenneth Kaunda district of the North West province.

## Methods

### Study setting

The study was conducted in Dr KK district in the North West Province of South Africa. The Dr KK district is the economic hub of the southern part of the province, and the healthcare facilities in this district are often prioritised for pioneering healthcare initiatives in the North West Province [19], this means the district is trusted by the National Department of Health for being early adopter in implementation. For example, the North West Provincial Health Department has been leading a social franchising project for primary healthcare, a government-led initiative currently piloted in this district [20]. Also, the district’s involvement in the NHI pilot programme further supports its proactive approach to improving healthcare services.

This study included one tertiary hospital and two secondary hospitals to provide a comprehensive understanding of PSI reporting practices in this district. Tertiary hospitals are central to highly specialised treatments and multidisciplinary care, whereas secondary hospitals provide specialised services unavailable at the primary care level, a larger percentage of provincial healthcare resources is supposed to be allocated to tertiary and secondary care levels [21, 22]. Additionally, these hospitals serve as clinical learning environments for the North West University (NWU) health science students, making it critical to support best practices and enhance their competency in patient safety.

### Study design

A qualitative, exploratory design was employed to explore implementers’ (healthcare providers’ and healthcare leaders’) views in implementing the national guidelines for PSI reporting in the hospitals in Dr Kenneth Kaunda District.

### Conceptual framework

This study was guided by a Policy Triangle Framework (PTF), developed by Walt and Gilson (1994), a well-established model for analysing complexities of health policy formulation and implementation [23]. The PTF examines policy through four interlinked dimensions: context, content, actors, and process [23] and has been widely applied in diverse health settings, demonstrating its adaptability and relevance for policy implementation research [24, 25].

**Fig. 1:**
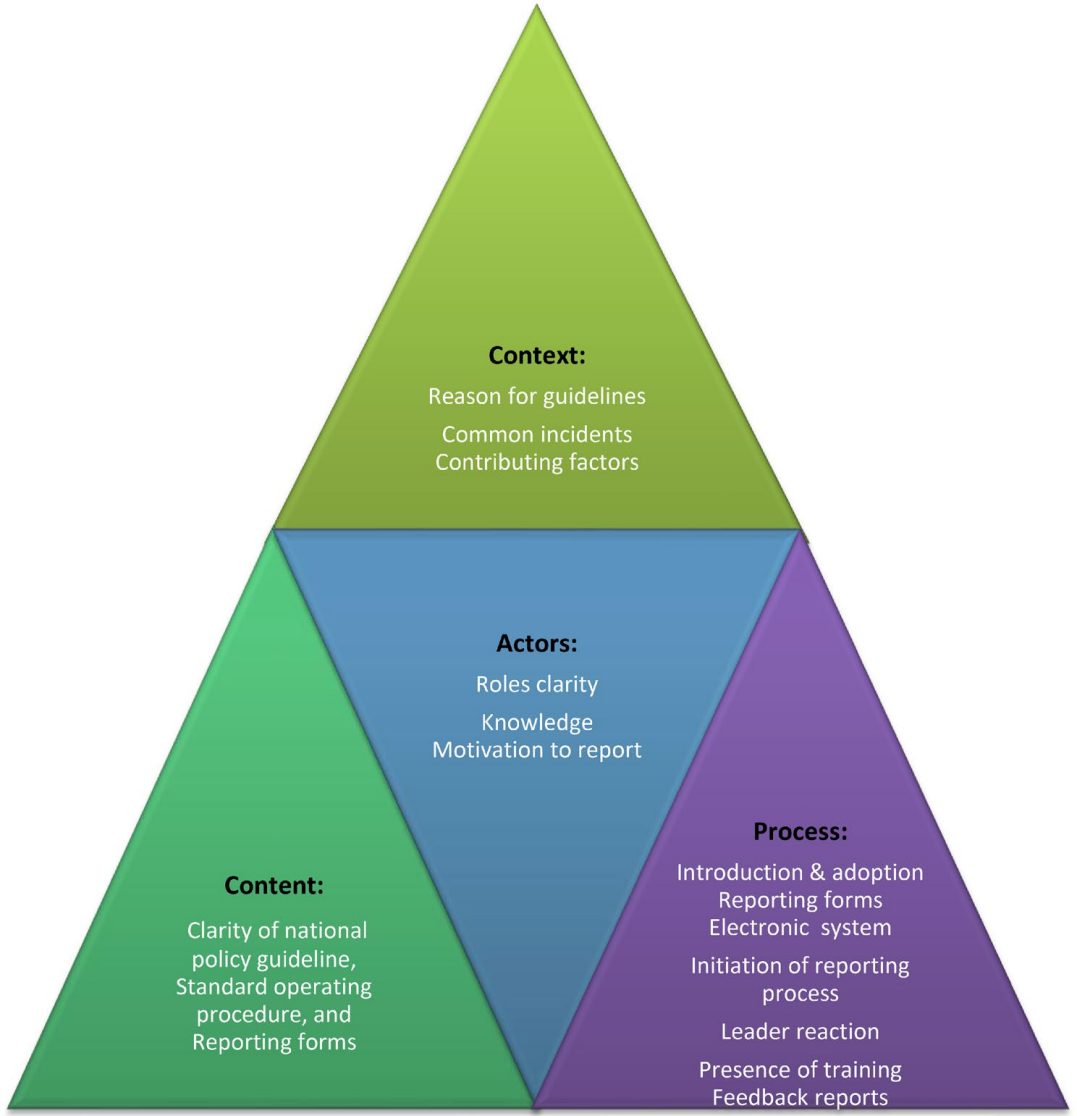
Policy analysis framework, adapted from the Policy Triangle Framework developed by Walt and Gilson [23]

In this study, the national guidelines for PSI reporting were considered as a national policy requiring implementation across all health facilities by designated policy actors, namely, hospital leaders and frontline healthcare providers. The PTF enabled a comprehensive exploration of how the guidelines were understood and implemented within hospitals. The context dimension examined the history of introducing guidelines and common PSIs and their contributing factors. **Content** focused on the clarity of the guidelines, including standard operating procedures and reporting tools. **Actors** referred to the individuals responsible for implementing the policy, their roles, responsibilities, knowledge, motivation, and behaviour recognised as determinants of implementation success. The **process** dimension focused on the steps of reporting, management of reporting, including feedback loop. Aligned with the study aim, the PTF guided the exploration of implementers’ perspectives across all four domains in implementing the national PSI guidelines. Figure 1, illustrates the adaptation of the framework.

### Population and Sampling

A purposive sampling of medical doctors, professional nurses, physiotherapists, pharmacists, occupational therapists, and hospital leaders was done. These professionals were selected for their direct involvement in patient care and in healthcare leadership, serving as primary actors in the implementation of PSI reporting guidelines. Since we had three participating facilities, in each facility we held focus group discussions with leaders and healthcare providers. Each FGD consisted of four to seven participants, saturation was not quick to establish due to variations in professional groups, we experienced saturation at each second last discussion in each facility and added an additional discussion to confirm saturation. We intentionally grouped participants with common characteristics of being all leaders of the same facility, or same professional category, this was to remove hierarchical barriers and to encourage perspectives in various professional categories. A total of eight focus groups in Hospital A, eight in Hospital B, and seven in Hospital C were held, leading to 23 FGDs during saturation point. Overall, the participating policy implementers were operational managers (*n* = 21), patient safety committee members (*n* = 10), doctors (*n* = 14), nurses (*n* = 35), pharmacists (*n* = 21), physiotherapists (*n* = 12), and occupational therapists (*n* = 10) across the participating hospitals.

### Data collection instrument

A semi-structured focus group discussion interview guide (see additional file 1) was developed in English, guided by Walt and Gilson’s policy triangle framework [23]. In addition to the section on demographic data of participants, the guide had questions focused on context, actors, content, and process of implementing the national guidelines for PSI reporting. The tool was reviewed by the research team for content validity and was piloted in one hospital in the province, which was not included in the study. There were no revisions required; specific attention was paid to the time it took to complete the discussion, the clarity of the questions asked, and the management of group facilitation.

### Data collection

Following the ethical approval and relevant permissions, data collection commenced between September 2023 and July 2024, using focus group discussions (FGDs) to collect data. It was ensured that each focus group consisted of participants of the same category, for example, not mixing subordinates with their managers, to encourage freedom of expression [26].

In each session of data collection, the researcher began by welcoming participants, outlining the study’s purpose, and emphasizing key ethical principles, including confidentiality, voluntary participation, and respect for diverse viewpoints. To maintain anonymity, each participant was assigned a unique code. Consent for interview and for recording was obtained, and all FGD participants signed a confidentiality agreement. The focus group discussions were led by SD using a semi-structured interview guide, while ST made notes. This approach ensured consistency across sessions while allowing flexibility for natural dialogue. The opening question invited participants to share common PSIs in their facility, which led to discussions of the context of the development of the PSI guideline, content clarity, actors’ roles, knowledge of policy, motivation to report, and process of implementation. A total of twenty-three (23) focus group discussions were conducted by the time saturation was reached, each lasting approximately 60 to 90 min. Each discussion concluded with a summary of key points, which participants reviewed and confirmed for accuracy, findings were shared with participants from each hospital for confirmation.

### Data analysis

The first author transcribed data, and both SD and ST listened to the audio recordings and read the transcriptions to validate the accuracy of the transcripts and correct any transcription errors. Upon completion of data cleaning, deductive thematic analysis was conducted using the steps outlined by Braun, Clarke, and Hayfield [27], in line with the policy triangle framework. For example, line-by-line coding was done, annotating initial thoughts alongside each line to generate initial codes. Noteworthy aspects of the data were systematically coded throughout the entire dataset. An additional examination of the sub-themes involved assessing their coherence with the coded extracts and the entire dataset [28]. Further analysis of the sub-themes was refined, the nuances of each sub-theme were examined, and the overarching narrative was aligned to the framework. The researchers discussed the codes until they reached an intercoder agreement [29]. The other two authors (AAA and NS) reviewed the analysis and confirmed the accuracy of the presented themes and sub-themes.

### Trustworthiness

We applied principles of trustworthiness by Lincoln & Guba [30]. **Credibility** was ensured in multiple ways, including data triangulation through seeking perspectives from leaders and various health professional category participants. Having data collection, and analysis done by both SD and ST contributed to prolonged engagement and researcher triangulation. Inclusion of participants who have been actively involved in implementing PSI reporting guidelines provided data accuracy. Findings were initially shared with participants from each hospital for confirmation, and subsequently, two dissemination meetings for each hospital included over 50 hospital leaders and frontline healthcare providers who also confirmed the truth of the findings. **Confirmability** was achieved through the use of direct quotes from participants, grounding the findings narrative on what was said by the participants. A clear description of the research objective, study setting, and context provided contributes to **transferability.** Alignment of research objective, interview guide, and clarity of data analysis approach enables **dependability**.

### Findings

As indicated in Table 1, there were 3 participating hospitals, named hospitals A, B, and C, to maintain confidentiality. A total of 123 participants participated in the study. Participants included operational managers (*n* = 21), patient safety committee members (*n* = 10), doctors (*n* = 14), nurses (*n* = 35), pharmacists (*n* = 21), physiotherapists (*n* = 12), and occupational therapists (*n* = 10) from all respective hospitals, each group being interviewed separately. Overall, the participants were predominantly female, middle-aged, highly experienced, and educated at a degree or diploma level, representing a diverse mix of professional categories across the three hospitals. Table 2 presents a summary of the research findings, themes, and sub-themes.

**Table 1:** Demographic data

Characteristics	Hospital A (n=38)	Hospital B (n=47)	Hospital C (n=38)	Total (n=123)
Age				
Mean age	39	40	45	
Gender				
Female, n (%)	31(81,57)	42(89,36)	35 (92,10)	108 (87,80)
Male, n (%)	7(18,42)	5 (10,63)	3 (7,89)	15 (12,19)
Experience				
0-5 yrs, n (%)	5 (13,15)	4 (8,51)	3 (7,89)	12 (9,75)
6-10yrs, n (%)	18 (47,36)	20 (42,55)	11 (28,94)	49 (39,83)
11-15yrs, n (%)	4 (10,52)	8 (17,02)	8 (21,05)	20 (16,26)
16-20yrs, n (%)	4 (10,52)	6 (12,76)	4 (10,52)	14 (11,38)
>20yrs, n (%)	7(18,42)	9 (19,14)	12 (31,57)	28 (22,76)
Education Level				
Diploma	9 (23,68)	18 (38,29)	14(36,84)	41 (33,33)
Degree	22(57,89)	23 (48,93)	17(44,73)	62 (50,40)
Post grad	7(18,42)	4 (8,51)	7(18,42)	18 (14,63)
Masters/PhD	0(0)	2 (4,25)	0 (0)	2 (1,62)
Participant Categories				
Doctors	5 (13,15)	4 (8,51)	5 (13,15)	14 (11,38)
Operational Managers	5 (13,15)	5 (10,63)	11(28,94)	21 (17,07)
Patient Safety Committee	4 (10,52)	6 (12,76)	0 (0)	10 (8,13)
Professional Nurses	9 (23,68)	14 (29,78)	12(31,57)	35 (28,45)
Pharmacists	6 (15,78)	9 (19,14)	6 (15,78)	21 (17,07)
Physiotherapists	4 (10,52)	4 (8,51)	4 (10,52)	12 (9,73)
Occupational Therapists	5 (13,15)	5 (10,63)	0(0)	10 (8,13)

**Table 2:** Summary of findings: Themes and Sub-themes

Category of the framework	Theme	Sub-themes
**Context**	• **Contextual issues leading to implementing guidelines**	• Standardising reporting practice • Improvement of record-keeping • Learning from the errors • Protection against litigation
	• **Common incidents & contributing factors**	• Identified incidents • Inadequate security response • Shortage of staff • Lack of resources
**Content**	• **Clarity of policy content**	• Clarity of National Policy Guidelines • Clarity of Standard Operating Procedures • Clarity of Reporting Forms
**Actors**	• **Role clarity**	• Discrepancy between expected and practiced roles • Clear roles of managers • No structured approach for reacting to reports
	• **Actors’ Knowledge**	• Knowledge strengths and gaps
	• **Actors’ Motivation**	• Motivators or demotivators towards reporting
**Process**	• **The reporting process**	• Introduction and adoption • Reporting in the manual form • Reporting in the electronic system • Initiation of the reporting process • Leader’s reaction to reported incidents. • Presence of training • Feedback on reports

## Policy context

The findings regarding policy **context** referred to the issues leading to the implementation of guidelines, common incidents, and their contributing factors.

### Contextual issues leading to the guidelines

Participants across all three hospitals reported several cross-cutting contextual pressures that created a need for the national guidelines for PSI reporting. These included the need to standardize reporting practice, improve documentation, enable organisational learning from errors, and mitigate medico-legal risk. Participants linked the implementation of policy to the absence of reliable documentation to report incidents, which leads to an increase in litigation cases.

*We faced an increase in litigation*,* but we had no clear records of what happened; we needed a standardized approach in reporting and management of PSIs. We also needed to create a reporting structure*,* clearly define what needs to be reported*,* and reduce the incidents from recurring by learning from our mistakes* (Professional Nurse, Participant 01- **Hospital B**)

Participants described the implementation of guidelines as a way to ensure incidents are documented before they escalate into legal claims, reinforcing that documentation and legal protection were motivations behind policy implementation.

*I would say they [the National Department of Health] want to make sure that before things happen*,* that it doesn’t become a bigger problem [referring to litigations] so to prevent them from happening and learning from mistakes*, (Unit manager, Participant 03- **Hospital A**)

### Common incidents and their contributing factors

Patient falls, pressure ulcers, maternal deaths, and abscondment were reported as common incidences across all the participating hospitals. Participants were of the opinion that key contributors to the identified common incidents were staff shortages, high workload, a lack of resources and inadequate security responses. For example, a physiotherapist described how inadequate equipment contributed to patient falls, indicating that safety risks may be embedded in the physical care environment.

*The reason why the patients fall*,* number one*,* we have a very high demand for chairs in our hospital*,* and we don’t have suitable chairs for patients to sit on; they don’t have armrests* (Physiotherapist, Participant 01- **Hospital B**)

Similarly, medical doctors highlighted how resource constraints disrupt clinical procedures, showing how workflow interruptions may increase the risk of adverse events.

*….you want to insert an IC drain*,* you lack resources to perform the procedure*,* there is also no nurse to prepare a trolley for you*,* if you missed something you have to deglove and go find the item and come back and do the procedure* (Doctor, Participant 02 - **Hospital C**)

Nurses described how staffing shortages limited their ability to perform basic preventive measures, such as turning immobile patients, which contributes to pressure ulcers.

*Doctors prescribe patient turnings*,* and as nurses we know that bedridden and very ill patients must be turned two or four hourly*,* but due to burn-out*,* nurses end up not carrying out doctors’ orders due to lack of staff*, (Professional Nurse, Participant 03 - **Hospital A**)

Some participants commented regarding the contributing factors to abscondment which are due to institutional security limitations, highlighting the role of organisational systems in shaping patients’ safety outcomes.

*If there were more staff*,* they might have noticed earlier that she’s not coming back. And the same with the security*,* the doors that can’t lock from the outside. Because if she didn’t have the means to go out because the doors are locked at the times that they were supposed to be locked*,* or if the security patrol to see who’s there and doing what*,* then maybe we can prevent things like that from happening*, (Patient Safety Committee, Participant 04 - **Hospital A**)

## Policy content

Policy content referred to information in the national guidelines document, standard operating procedure, and the manual reporting form.

### Clarity of policy content

#### **Clarity in the national policy guideline**

All healthcare providers across all hospitals had no knowledge of the policy guidelines on PSI reporting, citing a lack of access to guidelines or being large documents as reasons for their lack of knowledge. Although, the patient safety committee members had insight into the policy content, they verbalized no specific areas to be improved.

*The policy has a lot of information that is difficult for us to grasp. It has a****lot of information***. *It does not just go straight to the* point (Unit Manager, Participant 01- **Hospital A)**

*The guidelines****are not accessible to us***; *hence*,* I don’t know how to classify an incident as a PSI or just a routine issue*, (Physiotherapist, Participant 03- **Hospital C**)

*“I have****never read****the guideline document; I have only read the reporting form* (Doctor, Participant 02, **Hospital B**)

#### Clarity in the standard operating procedure

The standard operating procedure was well known among healthcare providers and the patient safety committee in all participating hospitals, except among medical doctors. Participants held varying opinions about the SOP’s content. Frontline healthcare providers found the SOP to be clear but lengthy, which reduced their interest or available time to read it.

*The SOP is very clear. We understand it because we are very familiar with SOPs. So*,* it tells us what a PSI is*,* how to go about it*,* and who to report to. It’s very straightforward. It’s something that we can relate to very easily because it’s not a thick document like the one from the National Department of Health* (Unit Manager, Participant 01- **Hospital A**)

*I am not knowledgeable about the SOPs*,* those documents are lengthy*,* there is no time to read them* (Doctor, Participant 03- **Hospital C**).

#### Clarity in the reporting forms

There were various views on the clarity of forms; some participants believed the forms were clear, while others reported a lack of clarity on the classification of PSIs. Some participants in hospital B reported that the SOPs and the form were not correlating. The following quotes indicate clear reporting forms, unclear classification, and non-correlating documents.

*“The form is clear for now. It took us a long time to get acquainted with the form* (Unit Manager, Participant 01-**Hospital A**)

*“The form is not clear on the classification of abscondment*,* and which classification it belongs to* (Professional Nurse, Participant 02- **Hospital C**)

*“The SOP and the reporting form don’t correlate. It just feels like they are drilling those PSI things into our heads and giving us an SOP that we must know and understand*,* but I just don’t see how it is clear* (Professional Nurse, Participant 05- **Hospital B**)

## Actors’ roles, knowledge and motivation

We focused on **actors’** roles, knowledge, and motivation in the implementation of the national guidelines for PSI reporting.

### Role clarity

#### Discrepancy between expected and practiced roles

The theme of roles referred to the roles of healthcare providers and healthcare leaders. Every healthcare provider was clear about the hierarchy of the information flow and expectation; however, it was not happening. The participants verbalised that there was a skewed attention and pressure to report, which was emphasised to Professional Nurses, and it seemed that the reporting of incidents was a responsibility of the professional nurse, followed by the other professional categories. Reporting of incidents was not affirmed as everybody’s role, excluding participation of lower categories of nurses, such as enrolled nurses, or enrolled nursing assistants, as well as the security guards, these individuals are more likely to witness incidents due to their roles. Professional nurses verbalised that they observed that the skewed expectations of their end have been used by the other personnel to sabotage them.

The quotes below indicate how different healthcare providers expected professional nurses to report or opted not to report, so that someone else does.

*Most of the time*,* a lot of incidents are actually filled by the nursing staff*,* that’s what I’ve noticed*,* and like with the incident report*,* there’s very little that I’ve personally filled that they asked me to fill in; it was mostly on the doctor’s notes* (Doctor, Participant 01- **Hospital A**)

Physiotherapists, occupational therapists, and other healthcare providers often witness incidents but generally do not take responsibility for completing incident reports; they are more likely to wait for nursing staff to handle the formal reporting.

*When an incident happens in a ward that’s not our specific area*,* we are not always the ones to report on the incident. When a patient falls out of the bed in the ward*,* due to not being restrained*,* we’re not usually the ones to take the responsibility to report the incident* (Occupational Therapist, Participant 04- **Hospital A**)

Enrolled Nurses and Enrolled Nursing Assistants are generally not directly involved in the incident reporting process. They are not permitted to complete the incident reporting forms, even though they may be the first to witness the incident. Their involvement is more passive, serving as potential witnesses to incidents rather than active participants in the reporting process. One manager explained the reasons for not allowing enrolled nursing assistants and enrolled nurses to complete the PSI forms:

*Lower nursing categories must not fill in the PSI form. Remember*,* this is a highly rated document. You can go to court. So*,* what is written as a PSI report should be quality information. I mean*,* an ENA [ Enrolled Nursing assistant] will just start writing things that are not comprehensive enough*,* but a professional nurse will be able to compile a comprehensive report*, (Unit Manager, Participant 04- **Hospital C**)

#### Clear roles of managers

Regarding the roles of managers, they verbalised that their role was clear and described it as being able to teach about incidence reporting, motivate reporting, and respond positively to the reported cases.

*As a manager*,* my role is to ensure measures are in place to prevent incidents and to investigate and escalate when they occur* (Unit Manager, Participant 03- **Hospital C**)

#### No structured approach for reacting to reports

Managers confirmed that they had no structured approach to responding to reported incidents, nor did they have a structured approach to motivating subordinates to report. This contributed to inconsistent reactions to PSIs, as well as the negative experiences of healthcare providers when reporting incidents

*There are no clear strategies on how to respond to errors. No training of managers on how we should respond to errors* (Unit Managers, Participant 03- **Hospital C**)

### Actors’ knowledge

#### Knowledge strengths and gaps

This sub-theme focused on the actors’ knowledge regarding reporting. As noted under theme two on their perspective on content clarity, their lack of knowledge of the guidelines and SOPs and being only familiar with the reporting form was evident. The specific areas of the documents that were least understood were the classification of incidents according to severity codes and the decision tree, which, when well-known, lead to accurate decisions on whether an error constitutes a PSI, leading to reporting.

*Staff still need their manager’s guidance to report accordingly. One of the challenges is that there is a categorisation and classification challenge on the SAC [severity assessment codes]* (Patient Safety Committee, Participant 04- **Hospital C**)

A concern was that the knowledge gap was sometimes evident amongst the leaders.

*In terms of the classifications*,* I’m still trying to understand which incident falls under which category. You would not know whether it’s a two or a three*,* or where they fall under. I’m still trying to differentiate between the different SACs* (Unit Manager, Participant 04- **Hospital A**)

### Actors’ motivation

#### Motivators or demotivators towards reporting

In all the participating hospitals, healthcare providers verbalized their motivation to report incidents and to advocate for reporting as mostly based on their personal values, a feeling that they would not be able to sleep at night if they kept an incident a secret. In addition to this, their professional accountability served as a motivation and a deterrent to future similar incidents.

*If no one saw me having the incident*,* I will still report because I will not be able to sleep at night*,* I would rather report minor issues just to keep my conscience clean* (Physiotherapist, Participant 03- **Hospital B**)

Participants also mentioned factors that demotivated their desire to report incidents. These included a lack of active improvements from reported incidents and fear of consequences when reporting, especially since the electronic system sends information to the national level immediately. Most participants verbalized that for these reasons, they were not interested in reporting.

*I filled in hundreds of PSIs*,* but nothing has happened. The same PSIs I filled in three years ago; I’m still filling them in today. Nothing has changed. Those same incidents are still happening. So*,* you lose motivation* (Doctor, Participant 02- **Hospital B)**

*Sometimes the reason a patient fell is with the quality of bed they are sleeping on*,* the bed is broken*,* there is no lock*,* there is no budget*,* we can’t get a new bed*,* it is these kinds of problems that make you not bother reporting* (Doctor, Participant 02- **Hospital C)**

*Another thing that’s scaring us to report is facing the challenges*,* they will take us to a disciplinary hearing*,* and we will lose our jobs*, (Professional Nurse, Participant 02- **Hospital B**)

## Policy process

We focused on exploring the **process** of implementing the national guidelines for PSI reporting.

### The reporting process

The reporting process was about the introduction and actual implementation of guidelines.

#### Introduction and adoption

In hospitals A and B, participants verbalised that the guidelines were introduced as a coercive instruction to report incidents; otherwise, one would face litigation. There was an overemphasis on the negative consequences of not reporting, making learning a secondary priority of reporting. This has instilled fear amongst the healthcare providers and added a reason not to report.

*When the guidelines were introduced*,* the emphasis was on a need to document the incidence to avoid litigations*,* even now when managers find you not reported*,* the first thing she mentions is that*,* why you did not report do you want to go to jail. to make us report on time before litigation occurs* (Professional nurse, Participant 06- **Hospital A**)

The physiotherapists verbalised being introduced to the reporting form only when there is an incident, and that they have not discussed guidelines in their departments.

*I was only introduced to the PSI reporting form once I had an incident* (Physiotherapist, Participant 03, **Hospital B**)

Although there are clear expectations for the reporting process, there have been no efforts to create an atmosphere that encourages reporting.

*We are scared to have a PSIs in our wards*,* my colleague and I have already received a warning*,* because of patients developing pressure ulcers in our wards. The warning comes from senior management… it’s called consequence management* (Professional nurse, Participant 05- **Hospital A**)

#### Reporting in the manual form

In all three hospitals, the complexity and length of the form were reported as constraints, with healthcare providers often neglecting to report incidents altogether. Many expressed frustrations with the process, describing it as lengthy and overly complicated. In a setting where time is a scarce resource, filling out a PSI form becomes an additional burden, competing with immediate patient care priorities.

Reporting in the manual form is reportedly a tedious and time-consuming process.

*The form is a very long*,* complicated form*,* you know and we’re very busy. Now*,* to sit and to fill in that form*,* it’s a problem. We don’t have time for that. It’s not just*,* uh*,* a ten-second thing to fill in a PSI form. It takes some time to fill in a PSI form*,* and we have many more matters to get to*,* you know*, (Doctor, Participant 2- **Hospital B**)

In hospitals B and C, some participants reported that the reporting form had limited space, which restricted the information they could provide when reporting. Others improvised by attaching an additional page to create more space. Participants who observed a limited space for reporting had this to say:

*Normally*,* the Dr will write the PSI form on account 1*,* and I write on account 2; the challenge is that there is not enough space on the reporting form* (Nursing Manager, Participant 03- **Hospital B**)

#### Reporting in the electronic system

In addition to the manual form reporting, the data from these forms needs to be further captured into the electronic reporting system. Participants shared various experiences of the system. In hospitals B and C, the participants reported that the electronic reporting system was user-friendly, and the presence of data capturers who captured the information completed in the manual forms was useful; however, they incurred challenges with the network & passwords.

*The challenge is how we get to capture; we are dependent on the network. That sometimes passwords expire*,* you need to capture*,* so somehow there’s an interruption of reporting*, (Patient Safety Committee, Participant 04- **Hospital B**)

*We complete the form*,* and there is a data capture that captures the data on the system*,* this is very useful* (Professional Nurse, Participant 04- **Hospital C)**

In hospital A, the participants reported that the reporting is primarily done by hand, with only one staff member authorized to input into the system. Patient safety leaders reported that while the system was comprehensive, resource limitations often hindered its effectiveness.

*We complete the PSI by hand. We only have one place where they can capture it online*,* and only the quality assurance manager has a password to access the electronic reporting system* (Unit Manager, Participant 03- **Hospital A**)

#### Initiation of the reporting process

In hospitals B and C, doctors verbalised that they only reported when asked by a senior to report, and their reporting was not voluntary. Most healthcare providers reported that they were more comfortable reporting an incident in their notes than in the PSI forms and electronic system. This is because they fear exposing themselves at a national level, as these reports are automatically submitted at the national level, rather than being captured at the hospital level.

*If the consultants feel like something may have gone wrong in the management of a patient*,* they will say*,* Dr …*,* please fill in a PSI form for this case. And then you’d be handed a form*,* your senior gives you a form*,* and you fill it out. That comes with a lack of motivation. If your consultant doesn’t tell you to fill in a PSI*,* you won’t. And then now this becomes consultant dependent* (Doctor, Participant 02 -**Hospital B**)

Occupational therapists and Physiotherapists verbalised that their lack of clarity on what to report as incidents led to several occurrences not being reported outside their department, more so because they are not expected to report them.

*I feel like there are times when incidents happen*,* and no one takes accountability for it because they feel like the patient just fell. No*,* I was not in charge of this patient* (Occupational Therapist, Participant 03- **Hospital A**)

Pharmacists in all hospitals reported having their own exclusive internal approach to reporting minor incidents. The motivation for this is that they have experienced several incidents involving prescription errors between them and doctors, and they would rather resolve them immediately than report them. They mentioned that they turn these into internal lessons amongst pharmacists. While this may seem to be a good practice, it is not known how many of these incidents are minor.

*We don’t report errors on the national system; we have our own internal reporting system. We have a drug intervention file at our outpatient and inpatient sections within the pharmacy. Every pharmacist is aware of that drug intervention file*,* so if you find minor medication errors*,* we normally write them in there. We do a monthly compilation of all the forms and errors and present them at the quarterly PTC meeting* (Pharmacist, Participant 06- **Hospital B**)

There was a recurring preference among participants, especially nurses and doctors, to report on healthcare provider notes rather than using the national reporting system. Participants noted that practitioner notes are more immediately accessible, familiar, safer and easier to incorporate into their workflow compared to the national system, which may be seen as more complex or less user-friendly.

*I am comfortable with writing in the nursing notes*,* not in the incident form* (Professional Nurse, Participant 03- **Hospital B**)*”*.

#### Leader reaction to reported incidents

In hospitals B and C, very few participants verbalised a positive reaction from their supervisors when they reported an incident. Some experiences that stood out was when they witnessed someone senior reporting an incident or being supported by a senior person during the reporting process.

*I have experienced support from the unit manager*,* she showed me how to complete the PSI forms* (Professional Nurse, Participant 03- **Hospital C**)

*I am very comfortable because of my seniors’ reaction to the errors. My senior finds a way to turn the error into a learning experience and discusses how to deal with it next time*, (Doctor, Participant 01- **Hospital B**)

Most participants, more especially nurses and medical doctors, reported being victimised and threatened when reporting a PSI, which subsequently led to suicidal thoughts and absenteeism. They verbalised that reporting of incidents became a reputational risk to their professional being, rather than being used as a learning tool.

*I have experienced negative confrontation by colleagues*,* when they picked an error which I have missed*,* and as much as it is good to know so I learn*,* there is a blame approach amongst one another* (Doctor, Participant 02- **Hospital C**)

*Even other people get so scared of reporting incidents because they’re scared of being victimized* (Professional Nurse, Participant 02- **Hospital A**)

#### Training on patient safety incident reporting

Participants verbalised that the training on PSI reporting has been more focused on nurses, and that it was insufficient and not continuous, not considering the high levels of staff turnover, which led to a lack of certainty whether the practicing healthcare providers are the ones previously trained. Medical doctors verbalised that the training times are not favourable for them and that they are often unavailable for face-to-face training that occurs during the day, due to their work priorities, coupled with a staff shortage.

*There is no quality learning that takes place in the routine reading of the SOP*,* even though there is repetition of reading. The repetition is leading to boredom* (Professional Nurse, Participant 05- **Hospital B**)

*We are too busy with work. If you expect us to dedicate time during our eight-to-four for training*,* it’s not going to happen. If you want to offer us training*,* it must be done after hours* (Doctor, Participant 02- **Hospital B**)

Physiotherapists and occupational therapists reported that training appeared to be disproportionately focused on nurses.

*I know the quality team goes to the wards to teach nurses about all new updates but never comes to the allied or the outpatients to give training about the national guidelines and the changes that were made* (Occupational Therapist, Participant 03- **Hospital B**)

Several healthcare providers mentioned that, despite the presence of training programs, the content was often too basic or not tailored to the specific needs of their roles. Nurses and pharmacists reported that training sessions are often ad hoc and inconsistent across different shifts and departments.

*There is training is on the SOPs*,* but training on national policies is minimal* (Pharmacist, Participant 03- **Hospital A**)

#### Lack of feedback on reported incidents

Participants raised concerns about the insufficient feedback on reported cases, which led to a lack of learning from these incidents. This absence of closure affected confidence in the system and the perceived value of reporting.

*When we fill in the PSIs*,* and we submit them*,* we rarely get any feedback or hear back from what happens afterwards. There are a few cases that go up to PSG (patient safety group)*,* but the PSGs are usually attended by more senior doctors. So*,* it seems like from a junior doctor’s standpoint*,* all we do is just fill in the form. Uh*,* so there’s not much motivation in filling those in because we don’t even know what happens to them or whether they yield any benefit*, (Doctor, Participant 02-**Hospital B**)

*I feel there is no point in me reporting something*,* and yet I don’t get feedback from the people who are requiring me to report*,* for them not to come back and give us feedback*,* it really puts you down* (Professional Nurse, Participant 02**-Hospital A**)

## Discussion

This study aimed to explore the perspectives of implementers on the implementation of national guidelines for PSI reporting in the selected hospitals in the Dr Kenneth Kaunda district of the North West province of South Africa, guided by a PTF of context, content, actors, and process. The use of the policy triangle tool demonstrated the interaction of actors with content, how the actors’ knowledge, motivation, role clarity, and experience enabled or constrained the implementation. The findings also demonstrated how actors’ perspective of context, and their experienced implementation processes, enabled or hindered the reporting of PSIs. Although the domains of the policy triangle framework are interrelated, below we discuss the perspectives of the implementers on each domain of the policy triangle, and highlight policy implementation enablers, gaps, and missed opportunities, in line with the literature.

### The policy context

According to Moat, Lavis [31], understanding the context in which a policy is implemented often provides crucial lessons in improving implementation and enhances the buy-in of implementers [32]. The implementers’ positive perspective of the need to have the guidelines for PSIs reporting is a strength and an enabler to the implementation of the national guidelines for PSI reporting. It demonstrates alignment of the implementers with the vision of the policy maker and was meant to facilitate buy-in and reporting. A major concern was their overemphasis on self-protection against litigation rather than learning, as a reason the guidelines were implemented. Within the context domain, it is evident that the hospitals had an ongoing culture of keeping long-standing identified risks unattended, such as an ongoing shortage of staff, lack of resources, failure to manage contributors to patient falls and abscondment, which has created a culture of despair amongst policy implementers. The findings advocate for a need to apply a systems thinking approach in managing the identified patient safety risks, including prevention of already identified risks, to encourage reporting [33]. A major missed opportunity is the utilization of the data on common incidences in these hospitals to advocate for resources, as such actions could build trust with implementers and encourage reporting. The trends of common incidents could also be used to inform the training of clinicians and non-clinicians (security guards) about their role in patient safety, allowing them access to report PSI directly to the online platform, so they take ownership of the reporting process.

### The policy content

Clear policy content is an enabler and the main communication mechanism by the policy maker on how the policy is intended to be implemented. Lack of clarity on policy content impedes implementation [34, 35]. While it is commendable that the implementers perceived the SOPs and reporting tools as clear, their clarity did not translate to their understanding of the reporting process, this could mean that the implementers lacked drive for self-empowerment using the available, clear SOPs and reporting tools. The perspective of misalignment between reporting tools and standard operating procedures is an opportunity for the participating hospitals to review these documents, guiding PSI reporting in their hospitals. A major contributor to such gaps is insufficient inclusion of policy implementers at the policy development phase, leading to a policy development process being completed with minimal input from the implementers [36, 37]. Similar gaps have been observed in Indonesia and South Africa [10, 38]. In Jordan, the limited clarity of policy documents also led to inconsistent incident reporting [39]. Lengthy documents are discouraged in policy development, as they discourage comprehension, as reported in the UK and Australia studies, where detailed protocols reduced user engagement [40]. Instead, concise summaries alongside comprehensive SOPs are recommended to improve usability [41]. Since policy implementation is an iterative process, it is important for the leaders in the hospitals to continuously evaluate the usability of the documents that guide the PSI reporting; their feedback would inform updates of documents and training to enhance clarity.

### **Actors’ roles**,** knowledge and motivation**

For successful policy implementation, clarity of policy content, actors’ roles, knowledge, and motivation, and the implementation process are key [23, 42]. Role clarity facilitates action on policy implementation, determines who acts on the policy, motivation influence efforts to implement or resistance, and knowledge determines capacity to implement [43]. The culture of shifting the reporting responsibilities to professional nurses, coupled with several demotivators to reporting, such as punitive reaction to reported errors, lack of feedback and action after reporting, and the reported lack of knowledge on reporting, as well as previous negative consequences from reported errors, are all strong barriers to reporting. Unless these barriers are addressed, the reporting of incidents will not improve in these facilities. Creating a reporting culture that emphasise everyone’s role in reporting will enable improvement in reporting [44]. Similar challenges have been reported in South Africa, Singapore, and Uganda, where role ambiguity and fear of consequences hindered incident reporting [45–47]. Shared accountability models, in contrast, have improved reporting in other contexts [48]. Training on patient safety has been shown to enhance knowledge across all professional categories [49], suggesting standardized training could address these gaps. Our study unveiled missed opportunities on empowering managers in creating a culture that supports reporting of incidents, which could include multi-disciplinary team building to enhance trust between teams, thereby enabling supportive environments. It is evident from our findings that the leaders need to cultivate a new culture which redefines the purpose of reporting incidents to that of learning rather than compliance to policy implementation. There are opportunities to build on the already existing positive values which some implementers verbalised that they would not live without reporting. A system to reward reporting could be devised, to influence a mind-shift that the most reporting team is not a poor-performing team, but a vigilant team. There are opportunities to report near misses rather than serious incidents to get teams used to reporting and learning from reported incidents. One of the seminal reports in patient safety science records that to err is human [50], and that medical errors are a resultant of multiple factors, than a single human factor, this is the mindset that will advocate for a positive reporting culture, while supporting professional practice. Punitive responses to errors are outdated approaches and are confirmed globally to discourage reporting and learning [2, 51], while organizations with non-punitive cultures have mitigated fear and promoted system-focused reporting [48, 52]. A more convincing approach to change of culture is role modelling of the non-punitive approaches, and vulnerability by seniors on their own errors, which could encourage a culture of transparency. A missed opportunity in these participating hospitals is the lack of engagement with existing resources; healthcare providers indicate the existence of policies and SOPs in their facilities, yet they confidently did not know the content thereof. In addition none of the implementers mentioned their engagement with the existing NDoH online course on reporting incidences [53]. Managers could encourage all staff members to undergo online training at their own time, and submit proof of training, this could be aligned with their performance management. Such an approach could ensure that everyone is empowered on incident reporting. Comprehensive, multidisciplinary, and practical training, combined with the integration of interactive techniques and early inclusion in undergraduate professional curricula, and in continued professional development, can enhance engagement and reporting competence [54–56].

### Policy process

A clear implementation process that is easy to execute is recommended for successful policy implementation, no matter how good the policy may be, if it fails implementation, it needs to be revisited [57]. The implementer buy-in largely depends on how they were introduced to the policy [57], and in this case, how the implementers were introduced to the reporting process seems to have contributed negatively, as they verbalised that they were coerced into reporting. This is another piece of evidence that there is a need for a culture shift regarding the professed purpose of reporting. The redundant duplicated effort in reporting manually and electronically is not enabling the realisation of this policy implementation, amidst staff shortage, reporting on manual and electronic forms needs to be discouraged; a new system allowing reporting on mobile phones anywhere will facilitate the reporting process. There is an urgent need to discourage the exclusive internal reporting of the pharmacist departments, which could limit learning from the incidents, and which also encourages hidden records, all their internal reporting could be reported online and reflect nationally as near misses. Such records could be used to shape training needs and also used as reference to applaud teams that prevented number of near misses. The internal reporting happening in the pharmacy departments are also an indicator of a culture of fear to report. Enablers to the reporting process are a supportive organisational culture, which have been intentionally enacted into laws encouraging protection of reporters, encouraging anonymous reporting, prioritising learning from reports, seamless reporting approaches, with an emphasis on real time feedback communication on outcomes of identified risks [58]. An investment in positive organisational culture will encourage voluntary reporting, without being initiated by managers [58, 59]. Strengthening information technology infrastructure to enhance electronic reporting, coupled with staff training, and establishment of a psychological safe environment for reporting has shown to mitigate reporting barriers [60]. Timely and constructive feedback is critical for maintaining engagement and improving the reporting culture [61, 62], most importantly managers should be empowered to apply just culture in response to reported incidences.

The main problem leading to this study was an observed policy implementation gap in implementing the national guidelines on PSI reporting evidenced by insufficient reports and lack of learning from incidences. The use of PTF enabled identification of gaps related to context, content, actor’s role clarity, knowledge, motivation, including the reporting processes, we identified possible constraints, enablers and missed opportunities, which could be leveraged to narrow the policy implementation gap. Several issues, such as punitive response to errors, demotivators to reporting, contributors to errors, and common incidents are universal experiences; however, nature of incidents such as abscondment, failure to affirm reporting as everybody’s responsibility, and misinterpreted motive for the policy as a protective mechanism against litigation rather than for learning purposes, are more unique to South African context. A key policy implementation lesson from this study is the interconnectedness of the domains of the PTF in implementing the PSI reporting guidelines, requiring implementers to strengthen the policy context, especially motive, content clarity, actors’ knowledge, role clarity, motivation, and processes, to enhance implementation.

### Limitations and strengths

This qualitative study examined three hospitals in the North West Province of South Africa. While the findings cannot be generalised, the insights apply to other low-resource settings. The use of focus group discussions (FGDs) could introduce a social desirability bias when participants are responding in the presence of their colleagues; however, the inclusion of diverse groups of professionals and leaders may have counteracted this limitation. Using FGDs had several strengths, such as providing rich, in-depth insights into participants’ experiences and perspectives. The policy implementation framework provided a systematic and comprehensive approach to analysing the implementation. To our knowledge, this is the first study in the North West province of South Africa to analyse the implementation of the national guidelines for PSI reporting. The findings of this study have been disseminated in all the participating hospitals, and some recommendations have already been implemented in the hospitals, through support by the research team, the dissemination discussions created an opportunity to strengthen patient safety in the district.

## Conclusion

To improve the implementation of the PSI, a system-wide approach is necessary; healthcare providers and leaders’ knowledge must be improved, strategies to motivate reporting must be explored, leaders must create environments conducive for reporting, including protection of the reporters, and improvements after every reported system-level weakness are mandatory in order to encourage reporting. If reporting is for learning, anonymous reporting should be emphasised. Reporting SOPs must be clear, and processes must be made easy, and consider available technology.

## Supplementary Information

Below is the link to the electronic supplementary material.

Supplementary Material 1

## Data Availability

The datasets used and/or analysed during the current study are available from the corresponding author on reasonable request.

## Abbreviations

Dr KK: Doctor Kenneth Kaunda
ENA: Enrolled Nursing Assistant
EN: Enrolled Nurse
FDG: Focus Group Discussion
HREC: Health Science Research and Ethics Committee
NDoH: National Department of Health
NRF: National Research Foundation
NWU: North West University
OHSC: Office of the Health Standards and Compliance
PSG: Patient Safety Group
PSI: Patient Safety Incident
PTF: Policy Triangle Framework
SAC: Severity Assessment Code
SOP: Standard Operating Procedure
UK: United Kingdom
WHO: World Health Organization
