# Supplementary material for: Policy implementers’ perspectives of the implementation of the national guidelines for patient safety incident reporting in selected South African public hospitals
Source: BMC Health Serv Res. 2026 May 5;26:639. doi: 10.1186/s12913-026-14623-x (PMC13147645; doi:10.1186/s12913-026-14623-x)
Supplement: Supplementary file 1 — Supplementary Material 1 [file 12913_2026_14623_MOESM1_ESM.docx]

# **ADDITIONAL FILE 1: FOCUS GROUP INTERVIEW GUIDE**

**PATIENT SAFETY INCIDENT REPORTING STUDY**

| **Section A: Demographic data**   1. Date of interview: _____________________ 2. Hospital Code: _____________________ 3. Group code ______ 4. Participant code ____________   **Demographic data**   1. Age <35 36-40 41-45 46-50 2. Gender Male  Female  3. Years of practice: 6-10 11-15 16-20 >20 4. Highest academic qualification________________________________ 5. Position _____________________ |
| --- |

**Section B:**

| Planning |
| --- |

Venue: ___________ Time: _______________

Number of group members: ____________

Notes taker: _______________

Interviewer:______________

Sitting arrangement: ______________________________

| Approach |
| --- |

Establishing rapport:

Participants to understand basic FGD rules:

- Confidentiality
- Freedom to participate
- There is no wrong or right answer
- Important of a discussion than one person’s response

**Tip to the interviewer:** guide discussions through the stated sub-sections in section c

**Section C:**

| **Main discussion points guiding the FGD** |
| --- |

**POLICY CONTEXT**

- Common types and causes
- Let’s talk about the context: What triggered the initial implementation of PSI guidelines?

**POLICY CONTENT** (National guidelines, SOPs, Forms)

- Content clarity: What is your opinions on clarity of the guidelines, SOPs & forms

**ACTORS:** (Knowledge, role clarity, motivation_

- Let’s discuss your enablement to report, your knowledge of how to report, clarity of your role, and your motivation to actually do it

**PROCESS**: (Manual & or electronically)

- Let’s discuss the reporting process from the first time it was introduced to you
- Follow through step-by-step,
- Reaction once reported
- Use of information from reported PSIs

How can reporting be improved in this hospital

THANK YOU VERY MUCH FOR YOUR PARTICIPATION
